# Surgical repair techniques, functional outcome, and return to sports after apophyseal avulsion fractures of the ischial tuberosity in adolescents

**DOI:** 10.1007/s00264-021-04959-w

**Published:** 2021-05-08

**Authors:** Raymond Best, Anorte Meister, Jochen Huth, Ulrich Becker, Malin Meier

**Affiliations:** 1 Department Orthopeadic and Sports Trauma Surgery, Sportklinik Stuttgart GmbH, Taubenheimstrasse 8, 70372 Stuttgart, Germany; 2 Department of Sportsmedicine, University of Tuebingen, Hoppe Seyler Strasse 5, 72074 Tuebingen, Germany

**Keywords:** Avulsion fractures, Adolescents, Ischial tuberosity, Outcome, Return to sports

## Abstract

**Purpose:**

Among juvenile apophyseal avulsion injuries of the pelvis in adolescents, fractures of the ischial tuberosity are rare but sustainably debilitating. Also because informations on surgical repair options are very sparse and so far limited to general reviews, reports of individual cases or heterogeous small case series, practitioners, patients and their parental environment still feel a comprehensible hesitation regarding operative treatment. Therefore we intended to investigate patient related outcome measurements and return to sports rates after different types of surgical intervention in an own case series, so far unprecendented in its size.

**Methods:**

Patient data of adolescents that underwent surgical intervention for a displaced apophyseal avulsion fracture of the ischial tuberosity between 01/2015 and 12/2019 in our institution were gathered. Patients were then evaluated using the hamstring injury specific Perth Hamstring Assessment Tool (PHAT). Furthermore the return to sports level in comparison to the particular pre-injury level was rated.

**Results:**

Eleven adolescents with an acute or chronic mean fragment dislocation of 3.3 cm (SD ± 1.7) underwent surgical intervention in the assigned period. The mean post-operative PHAT score was 86.9 (0–100, SD ± 11.9) and thus good to excellent. The majority of adolescents (10/11) was able to return to their pre-injury sports, whereas 63.6% achieved full or nearly full level.

**Conclusions:**

Surgical refixation or restoration of aphoyseal avulsion fractures of the ischial tuberosity result in good to excellent outcomes and return to sport rates, irrespective of the type of intervention. Here prompt diagnosis with a timely intervention seems more promising than delayed interventions in chronic cases. Beyond 1.5 cm of fragment displacement affected patients should be counselled for surgical intervention.

## Introduction

In adolescents, during sporting activities, sudden large tension forces through the musculotendinous units may overload the cartilaginous growth plates at the particular apophyses of the attached and loaded tendon [1–3]. Though on the whole still rare, potentially resulting avulsion fractures with dislocation of the apophyseal bone mostly occur around the pelvis [4], being accordingly debilitating.

**Fig. 1:**
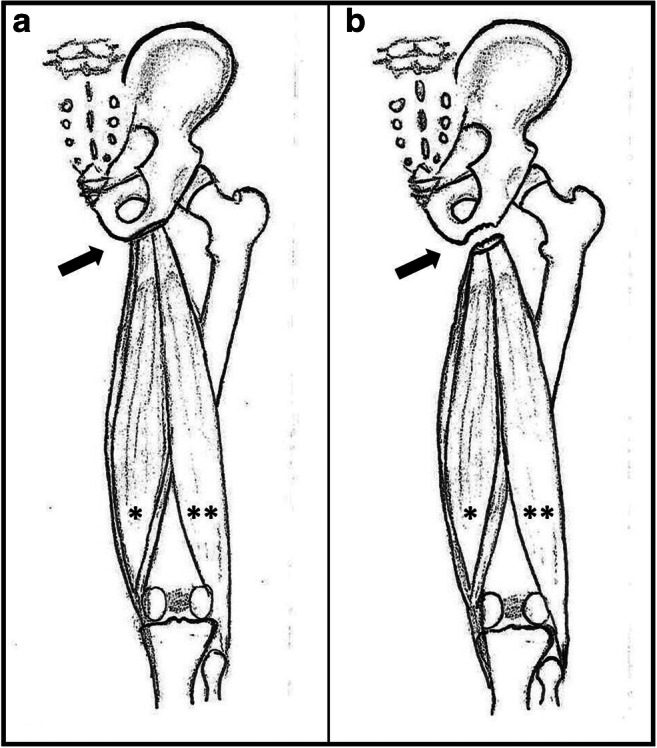
Schematic presentation of the attachment of the common hamstring tendon at the bony apophyseal ischial tuberosity (arrow) [a: regular situation, b: displaced apophyseal fracture with retraction and subsequent decreased muscular tension, star *: semitendinosus and semimembranosus muscle, two stars **: biceps muscle]

Commonly, the anterior inferior and anterior superior iliacal spine with fracture of the rectus femoris or the sartorius insertion respectively account for the majority of lesions [4]. However, in 10–30%, an apophyseal avulsion fracture may also affect the ischial tuberosity [2, 4] [Fig. 1]. Despite patients often report a clear crack in the pelvic region [2], not rarely without proper imaging the injury is misdiagnosed as pure muscular or musculotendinous injury [5–7].

Even in immediate and correct diagnosis, controversy exists regarding optimal therapeutic options [2]. Though limited, current literature describes surgery not to be necessary unless there exists a certain fragment dislocation [2–4, 8, 9]. Furthermore and naturally, responsibly deciding orthopaedic surgeons, young patients themselves and not least parental environment are often cautious with a decision for an initial surgical procedure. Additionally, the knowledge about apohyseal high healing potential in case of little fragment dislocation generally makes conservative treatment often favoured [9].

**Fig. 2:**
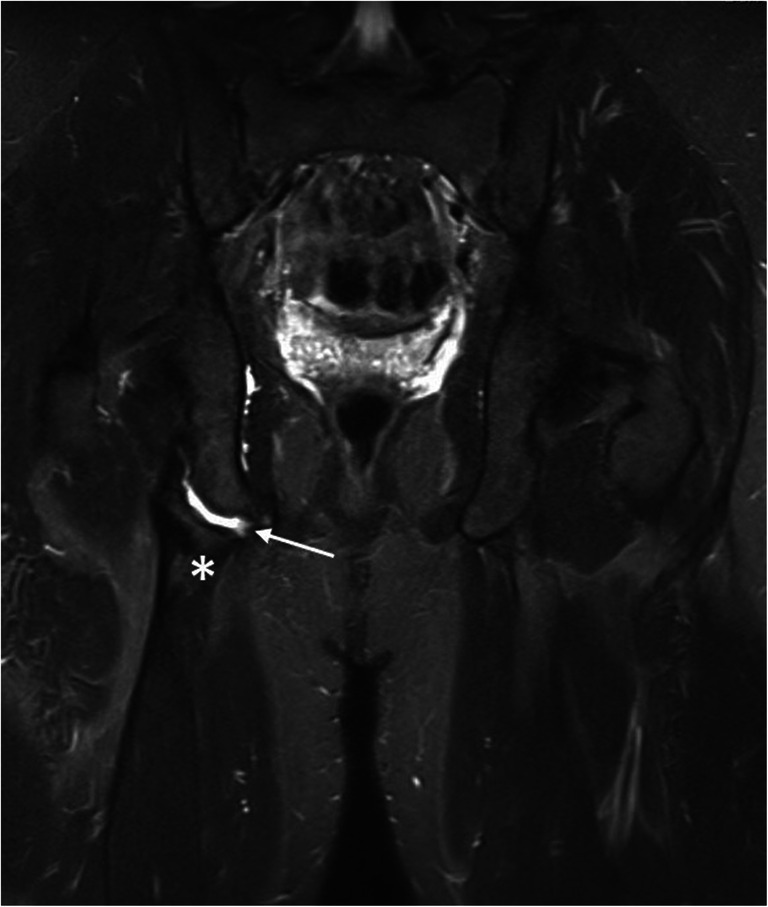
Non-union of a chronic avulsion fracture of a 17-year-old male adolescent 18 months after trauma (star *: apophyseal bone, arrow →: chronic non-union gap)

On the other side, previous singular case reports with one to three patients [1, 3] and studies of very small cohorts [6, 12] in part describe moderate outcomes of conservative treatment and consequently recommend to opt for surgical treatment in order to obtain best possible results. Here, it is argued that a delayed or even non-union of the ischial fragment with potential remarkable impairments should be avoided in any case [Fig. 2] [9].

To our knowledge, to date there is no study reporting on functional outcomes and return to sports in a singular larger case series of operated adolescents.

Therefore, the aim of our study was to particularly evaluate the functional outcome of hamstring avulsion fracture repair in an own case series of adolescents performing different surgical approaches. Here the Perth Hamstring Assesment Tool (PHAT) [5, 10], a validated, injury-specific, and meanwhile widespread PROM was used.

Besides describing the different operation approaches, we hypothesized that - irrespective of the type of surgery itself—any repair may lead to satisfying, good functional outcomes and good return to sports rates.

## Material and methods

For the presented case series, the data of all patients classified as proximal hamstring avulsion fracture between 01/2015 and 12/2019 in our hospital were gathered from our internal hospital database. For initial collection no differentiation was made between acute and chronic injuries as well as only patients with a maximum age of 18 at the time of trauma were included.

All medical records were reviewed, patient data and history, surgical documentation, and initial x-ray or and/or magnetic resonance images (MRI) were collected. For further inclusion, only patients with a clear apophyseal avulsion fracture and being skeletally immature (visible open epiphyseal growth plates) without doubt were included. Patients with closed growth plates or rather tendinous injuries despite an adolescent age were excluded from the survey.

Irrespective of the duration between trauma and surgery, at that time all included patients suffering from appropriate acute or ongoing symptoms and presenting with a fragment dislocation of more than 1.5 cm had been counselled for surgery. All MRI pictures were reviewed again in order to determine the stump retraction on MRI.

**Fig. 3:**
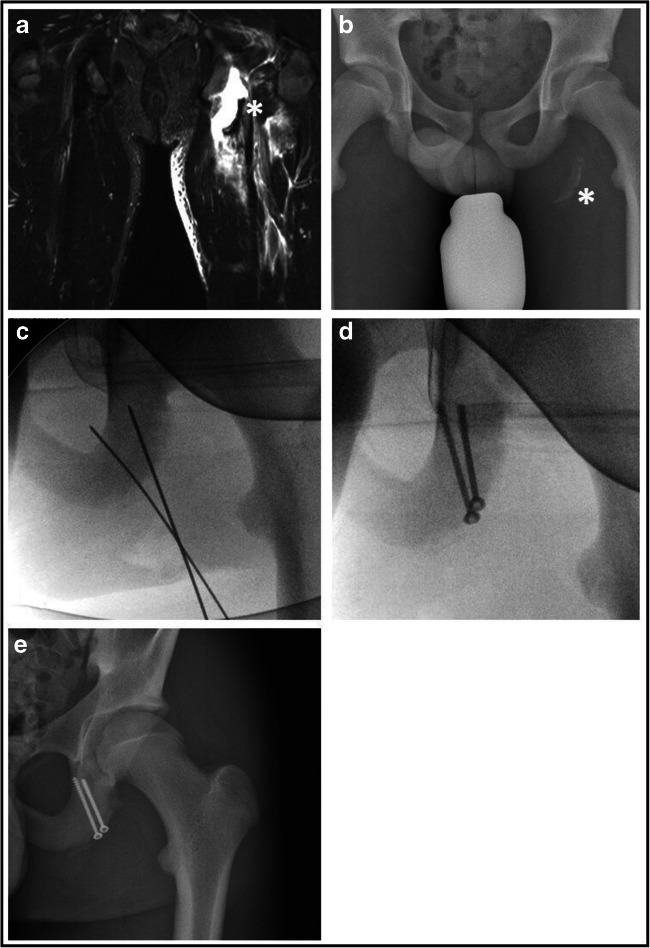
Refixation of acute left-sided avulsion fracture one week after trauma (a: coronal MRI picture, b: plain pelvis radiograph, c: intra-operative temporary fixation via guiding k-wires, d: intra-operative control after refixation with cancellous bone screws, e: post-operative control on plain radiographs, star*: bony fragment)

**Fig. 4:**
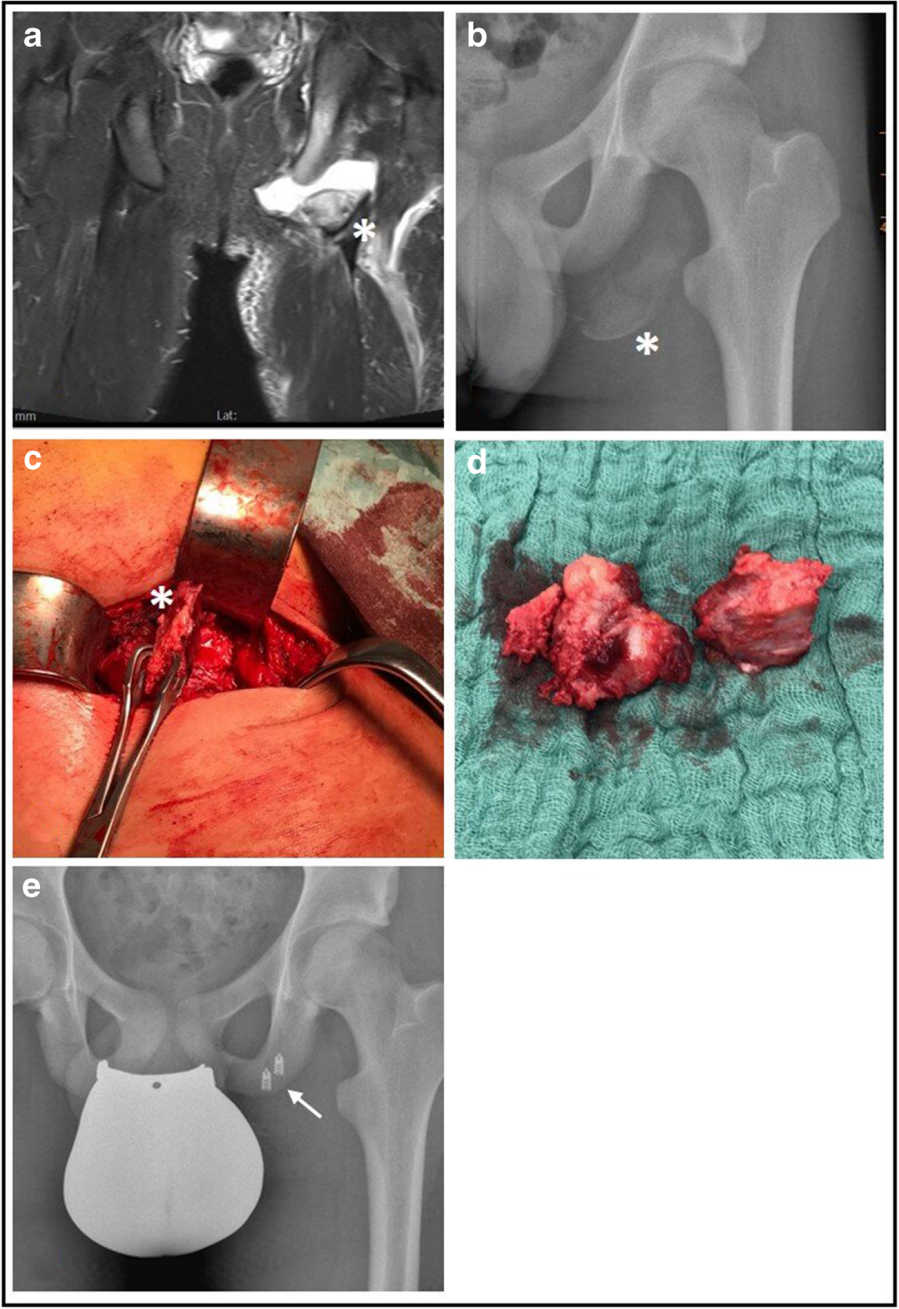
Excision of bony fragment in an acute left-sided avulsion fracture three weeks after trauma (a: coronal MRI picture, b: plain semipelvis radiograph, c: intra-operatively dissected bony fragment, d:excised bony fragment, e: post-operative plain radiograph control with refixated hamstring tendon, star*: bony fragment, arrow →: corkscrew anchors)

**Fig. 5:**
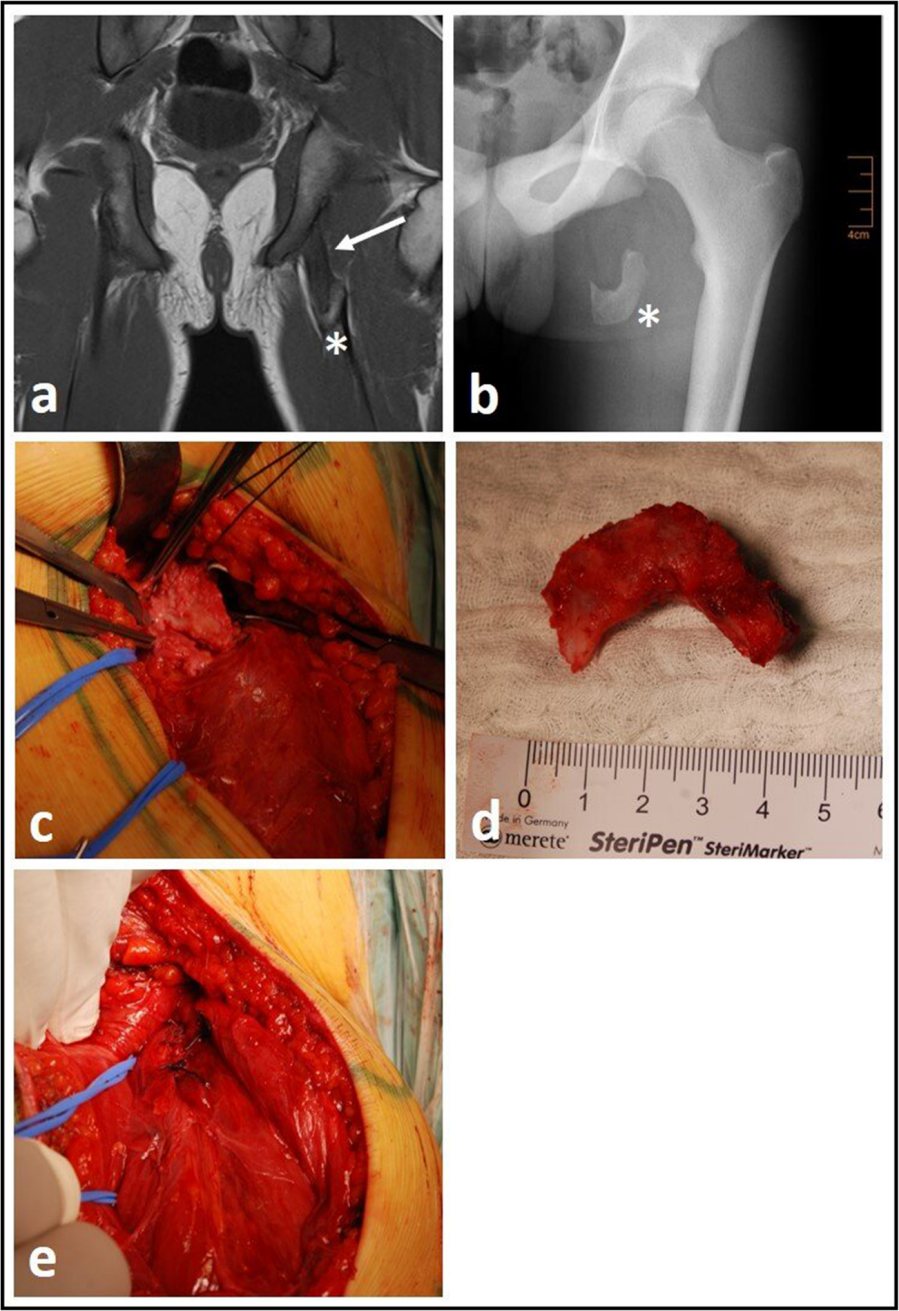
Excision of bony fragment in a chronic left-sided avulsion fracture one year after trauma (a: coronal MRI picture, b: plain semipelvis radiograph, c: intra-operatively dissected bony fragment, d: excised bony fragment, e: intra-operative end to end suture of tendinous scary plate, star*: bony fragment, arrow →: tendinous scary plate on MRI)

Depending on the time of surgery after trauma (acute versus chronic), the resulting distance of fragment displacement and not least symptoms related, one of the following three surgical approaches was chosen:

- Refixation of the apophyseal fragment by screws or anchors [Fig. 3a–e]
- Excision of the bony fragment and suture anchor repair of the tendons at the ischial tuberosity [Fig. 4a–e]
- Excision of the bony fragment and end to end suture of the tendinous scary bridge [Fig. 5a–e]

The repair itself was performed by two senior surgeons, author RB and coauthor UB of the study.

### Surgical approach

In all cases patients were situated in prone position. Surgery started with a longitudinal skin-incision of around 10 cm, beginning at the subgluteal fold. After superficial dissection, the gluteal fascia was incised, and potential haematoma (in acute cases) was evacuated. In acute cases with the absence of scar tissue, it was sufficient to visually identify the sciatic nerve, which was then held laterally in further course. In the majority of chronic cases (and if required due to the anantomic site), the nerve had to be carefully dissected from the attached tendon or bony fragment and was marked by a vessel loop for further preparation.

Beyond pre-operative considerations, the further operation procedure was then determined by the possibility of mobilisation of the apophyseal bony fragment. In case of good mobility with possible reattachment, the fragment was refixated. Here, the choice between screw and anchor refixaction was made upon the fragments size, vitality and stability. If the fragment lacked sufficient replacement possibility, it was excised. Hamstring tendons were then refixated at the footprint of the ischial tuberosity or readapted end to end to tendinous scary tissue.

The rehabilitation program was standardized as earlier described [11].

All included patients (and / or their parents) were contacted and asked for their informed consent to complete a questionnaire which was sent to them in further course, the Perth Hamstring Assessment Tool (PHAT). The self-administered and easy scoring system assesses the patients’ residual complaints and activity level during all-day and sportive activities.

Answers are then converted into a scoring system with a maximum score of 100 (maximum activity, no pain) and a minimum of 0 equalling the opposite. All returned and completed forms were statistically evaluated.

Furthermore, all patients were asked about their preferred sports prior to the injury. In addition to the completed forms, patients had to specify their level of return to preinjury sports.

Descriptive statistics was calculated for all registered scores. Results of all scores allowed accuracy to one decimal place.

Data was gathered and sorted using Excel 2016 (Microsoft, Seattle, WA, USA), while SPSS 24.0 (IBM, Armonk, NY, USA) was used for statistical analysis. Due to the comparably small sample size from a statistical viewpoint, figures and results were only calculated descriptively, correlations or else were not calculated.

## Results

In total, during the assigned period, 11 adolescents were surgically treated in our institution and could be included into the study.

The mean age at the time of trauma was 14.7 years (SD ± 1.8). Of the 11 patients ten were male, one female. The mean duration between trauma and surgery was 14.8 weeks (± 24.9), whereas the majority of patients (median) was operated upon within three weeks after trauma. Means and standard deviations of the single assessments as well as median values are displayed in Table 1.

**Table 1:** Overall data of patients, diagnostics, treatment option, PHAT outcome measurements and Return to sports rate (Ex: excision, Rev (A): refixation by anchors, Rev (S): refixation by screws, Tr: tendon repair)

**Patient No/sex**	**Age at trauma (years)**	**Time injury to surgery (weeks)**	**Stump retraction on MRI (cm)**	**Surgical technique**	**Follow up (months)**	**PHAT Score**	**Preinjury sports**	**Return to preinjury sports (%)**
**1 M**	15	56	7	Ex + Tr	51	76	Athletics	90
**2 M**	14	4	3	Ref (A)	40	89	Athletics	95
**3 M**	17	4	5	Ex + Ref	28	83	Soccer	95
**4 M**	15	3	2	Ex + Ref	17	96	Athletics	97
**5 M**	18	2	4	Ex + Ref	15	100	Soccer	100
**6 M**	15	2	3	Ref (A)	13	69	Soccer	80
**7 M**	15	72	1.5	Ex + Ref	10	67	Soccer/athletics	0 / 85
**8 M**	11	1	2	Ref (A)	9	97	Soccer	80
**9 M**	13	16	4	Ex + Ref	8	92	Soccer	75
**10 M**	14	1	3.5	Ref (S)	8	87	Soccer	90
**11 F**	15	2	1.5	Ref (A)	7	100	Dance	100
**Mean**	14.7	14.8	3.3		18.2	86.9		
**SD**	± 1.8	± 24.9	± 1.7		± 14.7	± 11.9		
**Median**	15	3	3		13	89		

The mean follow-up of all returned questionnaires (Return rate 100%) was 18.2 months (SD: ± 14.7 months). Mean total PHAT-score (0-100) was 86.9 (SD: ± 11.9) points.

The majority of patients (64 %) described soccer to be their preferred preinjury sports. With one exemption all adolescents participated in their preinjury sports, whereas more than half (6 patients) only had little impairments (> 90% preinjury level) and two described to have returned to an unlimited 100 % [Table 1].

Four patients depicted less than 90% of pre-injury level of which two adolescents had not received an acute but a delayed surgical intervention (> 3 months) due to an initial misdiagnosis of a muscle fibre injury [Table 1]. One patient with the longest operation delay (No. 7) had stopped with his pre-injury sports soccer and changed to athletics due to several reasons among which occupational ones. Though still not being completely pain-free after surgery during particular athletic activities, he nevertheless claimed to be satisfied in comparison to his pre-injury situation.

## Discussion

The most important finding of our study was that—irrespective of the type of technique—a surgical intervention after an avulsion fracture of the ischial tuberosity in adolescents with a fragment displacement > 1.5 cm leads to very good functional results with a subsequent high return to sports rate. Secondly it seems that an acute surgical intervention leads to better functional outcomes in comparison to delayed procedures.

To the best of our knowledge, this is so far the largest case series of avulsion fractures of the ischial tuberosity only focussing on surgical treatment as well as on the associated functional outcomes using an injury specific outcome score. Here the PHAT score mostly revealed very good to excellent results after surgical intervention with a high return to sports rate on preinjury level. Though the number of cases still does not justify any statistical correlation yet, it seems obvious that the lower return to sports rate of patient nine and especially seven might be associated with their delay between time of injury and surgery. Both patients received their surgery more than three months after trauma and need thus be regarded as “chronic” injuries [11].

Of course it can be argued that the subjective assessment of sports performance and PHAT score might be individually differing. For example in our patients, two soccer players rated their PHAT score almost equivalent [Patient 6+7] but described their further sports career differently. And of course are high pivoting sports such as soccer not comparable to mostly unidirectional sports such as athletics. Nonetheless our results confirm previously taken assumptions of better functional outcome results in case of prompt surgical intervention after trauma [2, 6, 12].

All in all our findings are in accordance with the sparse previous literature, also describing surgical intervention to be a good option in the named injuries and also concluding that surgery should be performed as soon as possible after trauma [6]

So far other descriptions about outcomes in avulsion injuries of the ischial tuberosity are limited to case reports [1, 13, 14] and heterogenous case series [3, 6, 12] and thus lack a clear recommendation or even a general consensus.

Just recently a perpetual systematic review and meta-analysis on clinical outcomes of apophyseal avulsion fractures in common [2] included 14 eligible studies with a total of only 29 injuries of the ischial tuberosity which confirms the rarity of adequate and seminal information on this topic in literature. Though other retrospective reviews in part describe higher numbers [4, 15, 16] their collection of data mainly focussed on prevalence, location and fragment description without respecting treatment options or outcome measurements.

However, regarding the few appropriate studies on outcomes so far, the majority of authors regard operative treatment as the method of choice in cases of significant fragment displacement. Here the benchmark is set between 1 [10] and 1.5 cm [2, 6]. Of course conservative treatment in patients with a fragment displacement beyond this named benchmark may also lead to good results, though the risk of protracted complications and complaints increases due to an uncontrolled healing. For example, using clinical aspects as well as the modified Harris Hip Score in 13 avulsion fracture injuries, Ferlic et al. [2014] described only 50% success rate of conservative management in four patients with a fragment displacement > 1.5 cm. Whereas two of these four patients had an excellent outcome without restrictions during sports activities, the remaining two patients developed a pseudarthrosis associated with occasional pain [6]. Both declined a recommended surgical intervention to relief the complaints.

In their systematic review and meta-analysis, Eberbach et al [2017] also concluded that patients with a fragment displacement greater than 1.5 mm and with high functional demands experienced a higher overall success and return to sports rate if they had received surgery [2]. Consequently they plead for a correct and timely diagnosis and treatment choice in order to avoid potential complications such as non-unions, heterotopic ossifications [6, 15] or even chronic mechanical sciatic symptoms due to mechanical irritations such as patient one of our study experienced.

Depending on the time between trauma and surgery as well as on the size of the bony fragment, several techniques have singularily been described to address the injury. Open reduction and refixation of fragment by screws or anchors [1, 13] seem desirable in order to aim for best possible muscle tension and anatomy. In case of a small or biologically insufficient fragment, an excision of the bony part and subsequent refixation of the tendinous stump is optional [3]. Of course both named techniques require an acute situation as chronic cases after months might not allow an appropriate redressing of the retracted fragment or stump.

In our study all described techniques were applied in dependance of the particular anatomical situation. Here none of the procedures revealed to be superior over another. We therefore conclude that even acute surgical treatment should not be limited to one special technique but that all described options should be controllable by the responsible surgeon in order to be able to choose the best particular appropriate option.

**Fig. 6:**
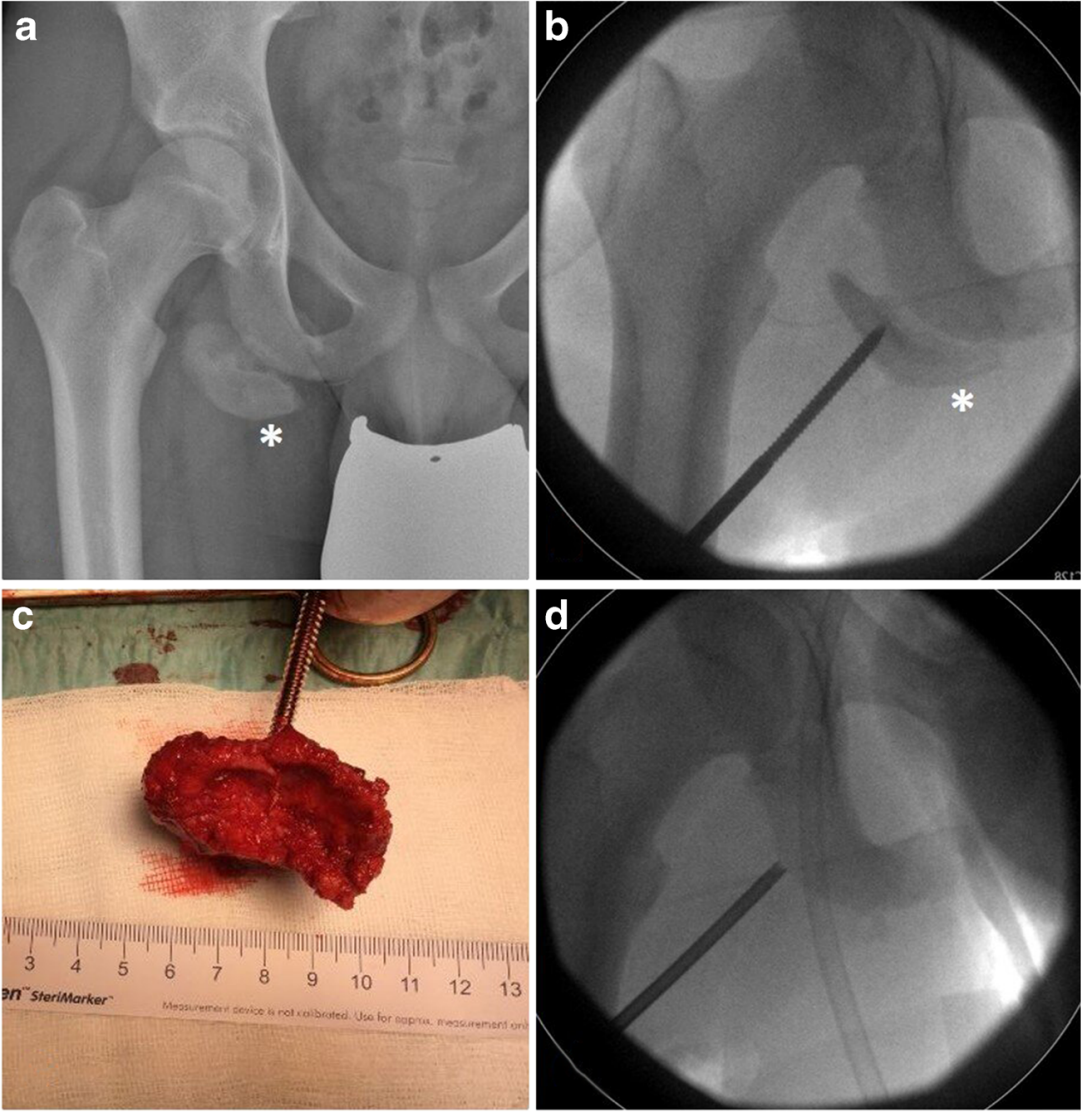
Hypertroph bony fragment in a chronic right-sided avulsion fracture 1 ½ years after trauma (a: plain semipelvis radiograph, b: attempt of anatomic repositioning of dissected bony fragment, c: excised bony fragment, d: intraoperative control of subsequent anchor placement for tendon refixation, star*: bony fragment)

Chronic cases are surgically even more demanding, requiring a costly neurolysis of the sciatic nerve. In further course the often hypertrophed retracted fragment [Fig. 6] mostly needs to be excised with a subsequent readaption of the hamstring tendon to either surrounding tissue or a scary bridge [patient 1].

In total three of our cases where operated in a chronic situation. As described previously by Sikka et al. in three cases [2013], pain could be relieved and normal strength could be achieved. Nonetheless none of our patients achieved a full PHAT score or a full return to sports, respectively. We therefore agree with their earlier conclusion [3] that a patient counselling for surgical treatment in chronic cases should include a possible improvement of specific complaints (such as sciatic symptoms due to mechanical irritation) but should certainly also respect a patients functional goal.

Of course, there are some limitations of our study.

First of all, though unprecedented in its size, the presented cohort is still very small, not allowing significant statistical conclusions and thus is of limited scientific expressiveness. Furthermore our results were not compared with conservatively treated patients and thus only allow a descriptive character limited to surgical treatment. However, as previous smaller case series could not yet clarify the controversy about optimal treatment, our study primarily intended to substantiate previous assumptions on time and type of surgery in case of an adequate fragment displacement.

Secondly the subjective assessment of the level of return to pre-injury sports at this age might underlie multifactoral causes and is not always caused by the trauma alone.

Not least we performed a retrospective study with all its inherent limitations such as an inconsistency of follow up or age. However, given the rarity of this injury, a prospective evaluation still is desirable but difficult to perform with adequate numbers. Furthermore, mainly the differentiation between conservative and operative treatment in relation to a fragment retraction and the time of surgery would be of superior interest. Here previous studies also of tendon avulsion injuries have already proposed a best possible prompt intervention if a stump/tendon retraction is more than 1.5 cm [17, 18].

## Conclusion

In conclusion, surgical refixation or restoration of apophyseal avulsion fractures of the ischial tuberosity results in good to excellent patient-related outcome measurements and return to sport rates, irrespective of the type of intervention. It seems that a prompt diagnosis with a timely intervention seems more promising than delayed interventions in chronic cases. Despite a comprehensible reservation of adolescent patients and parental environment, affected patients with an appropriate physical demand should therefore be counselled for surgical intervention if the fragment dislocation exceeds 1.5 cm.

## Data Availability

The datasets generated or analysed during the current study are included in the published article as well as they are available from the corresponding author on reasonable request.
